# Clinical Validity of Shear Wave Elastography for Post-Stroke Spasticity: A Systematic Review and Meta-Analysis

**DOI:** 10.3390/jcm15052063

**Published:** 2026-03-09

**Authors:** Ji Hyun Kim, Sen Jay Oh, Seo Young Kim, Tae Uk Kim, Yuna Kim

**Affiliations:** Department of Rehabilitation Medicine, College of Medicine, Dankook University, Cheonan 31116, Republic of Korea; jh0607@dkuh.co.kr (J.H.K.); sjaiz0901@dkuh.co.kr (S.J.O.); syoungrm@dkuh.co.kr (S.Y.K.); magnarbor@dkuh.co.kr (T.U.K.)

**Keywords:** stroke, spasticity, shear wave elastography, ultrasonography, muscle stiffness, Modified Ashworth Scale, Modified Tardieu Scale

## Abstract

**Background/Objectives**: Shear wave elastography (SWE) has emerged as a quantitative imaging technique for assessing muscle mechanical properties and has been increasingly applied to post-stroke spasticity. However, the clinical validity of SWE relative to established clinical spasticity scales and the influence of assessment protocols remain incompletely understood. This systematic review and meta-analysis aimed to evaluate the clinical validity of SWE for post-stroke spasticity and to identify clinically relevant methodological moderators. **Methods**: A systematic literature search was conducted in PubMed, Cochrane Library, CINAHL, and Web of Science to identify studies reporting correlations between SWE measures and clinical spasticity scales in individuals with stroke. Random-effects meta-analyses were performed using robust variance estimation to account for dependent effect sizes within studies. Prespecified subgroup and meta-regression analyses examined potential moderators, including clinical scale, muscle position during assessment, output metric, limb segment, and stroke chronicity. **Results**: Ten studies involving 303 participants contributed 38 correlation estimates. The pooled correlation between SWE and clinical spasticity scales was moderate (*r* = 0.42, 95% CI 0.34–0.49). SWE demonstrated significantly stronger correlations with the Modified Tardieu Scale than with the Modified Ashworth Scale. Measurements obtained in stretched muscle positions showed higher validity than those obtained at rest. Other examined moderators were not statistically significant. No evidence of publication bias was detected. **Conclusions**: SWE shows a moderate association with clinician-rated spasticity scales and appears to reflect the mechanical consequences of post-stroke spasticity. Associations were influenced by scale selection and measurement position. These findings support protocol-informed integration of SWE as a quantitative adjunct for assessing passive muscle stiffness rather than as a replacement for established clinical scales.

## 1. Introduction

Post-stroke spasticity is a common and disabling positive motor sign within the upper motor neuron syndrome [1,2]. It is typically described as a velocity-dependent increase in resistance to passive stretch and can contribute to pain, contracture, and limitations in daily activities [2,3,4]. In clinical practice, accurate quantification is important because relatively small differences in severity or muscle distribution can influence treatment selection and timing, including injection planning, therapy goals, and expectations of response [4,5,6].

The Modified Ashworth Scale (MAS) and the Modified Tardieu Scale (MTS) are the most widely used tools for assessing post-stroke spasticity in rehabilitation settings, but both are limited by subjectivity and examiner dependence [4,5,6]. MAS provides a single ordinal grade based on perceived resistance during passive movement, whereas MTS attempts to better capture the velocity-dependent component by standardizing stretch velocity and differentiating the “catch” angle (R1) from passive range (R2) [4,6]. Despite their clinical usefulness, measurement variability is substantial—especially in mild spasticity or when multiple muscle groups are assessed in one session—leaving ongoing debate about which clinical scale is the more appropriate reference when evaluating quantitative measures of spasticity [5,6].

Shear-wave elastography (SWE) has been increasingly studied as an ultrasound-based, quantitative method to measure muscle stiffness and may complement these clinical scales by providing device-generated values [7,8,9,10,11]. SWE estimates stiffness from shear-wave propagation within muscle and typically reports results as shear-wave velocity (m/s) or elastic modulus (kPa) [7,8,9,10,11]. The conversion between shear-wave velocity and elastic modulus (E = 3ρv^2^) assumes isotropic and linearly elastic tissue behavior [7,8]. However, post-stroke muscle undergoes structural remodeling—including fibrosis, altered fiber orientation, and architectural reorganization—which may violate these assumptions and potentially influence derived stiffness estimates [3]. Because prior studies variably report SWE outcomes in either m/s or kPa, and because structural alterations after stroke may differentially affect these derived metrics, it remains unclear whether one output measure more consistently reflects clinically assessed spasticity.

Beyond these theoretical assumptions, SWE measurements are also sensitive to practical testing conditions, including joint angle, passive muscle length, region-of-interest placement, and whether the muscle is assessed at rest or in a stretched position (STRETCH vs. REST) [12,13,14,15,16]. As a result, reported relationships between SWE and clinical spasticity scales vary widely across studies, making it unclear under which conditions SWE most meaningfully reflects clinically assessed spasticity [17,18,19,20,21,22].

Prior reviews that included mixed neurological and musculoskeletal populations have summarized this literature [17,18,19,20,21,22], but two issues limit direct clinical translation to post-stroke care. First, many primary studies report multiple correlations from the same cohort, and these correlated results can bias precision if the dependence is not addressed [23,24,25,26]. Second, several protocol-related questions that matter in routine rehabilitation—such as whether validity differs by clinical scale (MTS vs. MAS), by measurement position (STRETCH vs. REST), by output metric (m/s vs. kPa), by limb segment (upper vs. lower), and by stroke chronicity (subacute vs. chronic)—have not been evaluated using statistical methods that appropriately account for correlated effect sizes, despite their direct relevance to clinical implementation [7,8,9,10,12,13,14,15,16,17,18,19,20,21,22].

To address these gaps, we conducted a systematic review and meta-analysis in adults with stroke to (i) estimate the pooled correlation between SWE-derived muscle stiffness and clinical spasticity ratings using robust variance estimation to appropriately handle dependent effect sizes, and (ii) quantify the five protocol-related factors above to provide practical, evidence-based guidance for integrating SWE as a quantitative adjunct to routine spasticity assessment. Through this analysis, we identify key protocol-related factors that influence the magnitude of SWE–spasticity associations and provide evidence-based guidance for optimizing clinical implementation.

## 2. Materials and Methods

### 2.1. Protocol and Reporting

This systematic review and meta-analysis was conducted in accordance with the Preferred Reporting Items for Systematic Reviews and Meta-Analyses (PRISMA) 2020 guidelines [27] (Supplementary Table S6). The study protocol was prospectively registered in the PROSPERO database (CRD420251232085).

### 2.2. Data Sources and Search Strategy

We searched PubMed/MEDLINE, Web of Science Core Collection, CINAHL, and the Cochrane Library from database inception to December 2025. Search strategies combined controlled vocabulary and free-text terms for stroke, spasticity, and shear wave elastography and were adapted to each database. No language or publication-date restrictions were applied during searching. We additionally hand-searched reference lists of included studies and relevant reviews. The complete search strategies are provided in Table S1 [18,19,20,22].

### 2.3. Eligibility Criteria

We included observational studies of adults with stroke (subacute or chronic) in which (i) SWE was performed in paretic skeletal muscle and (ii) spasticity was assessed using the MAS and/or MTS. Studies were eligible if they reported, or provided sufficient information to derive, a correlation coefficient (Pearson or Spearman) between SWE outputs (shear-wave velocity in m/s or elastic modulus in kPa) and a clinical spasticity scale. We included cross-sectional studies and baseline (pre-intervention) data from clinical trials. We excluded non-stroke populations; animal, cadaveric, or phantom studies; case reports; conference abstracts without extractable quantitative data; and reports without an SWE–scale association.

### 2.4. Study Selection and Data Extraction

Two reviewers (J.H.K. and S.J.O.) independently screened titles/abstracts and full texts and independently extracted data. Extracted data included participant characteristics; limb segment and target muscles; assessment position and joint angle when reported; ultrasound system and vendor; SWE output metric; clinical scale and components (e.g., MTS R1, R2, and derived indices); correlation type; and numerical results. When a study reported multiple eligible correlations from the same cohort (e.g., across muscles, positions, scales, or metrics), we retained all eligible correlations and treated them as dependent effects within a study-level cluster rather than selecting a single outcome or averaging effects [23,24,25,26].

### 2.5. Risk of Bias Assessment

Construct-validity-related risk of bias was assessed using the Consensus-based Standards for the selection of health Measurement Instruments (COSMIN) by two reviewers, with disagreements resolved by consensus. We operationalized nine prespecified construct-validity checks (H1–H9) and summarized overall study quality using the worst-score-counts rule [28]. Given that SWE outputs are device-generated, unreported assessor blinding was rated as Adequate, whereas explicit non-blinding was rated as Doubtful. For the COSMIN precision item, sample size thresholds were prespecified as Adequate (≥30), Doubtful (20–29), and Inadequate (<20) [28].

### 2.6. Effect Size Calculation and Statistical Analysis

Correlation coefficients were transformed using Fisher’s *z* for meta-analysis and back-transformed to r for reporting. Sampling variance for Fisher’s *z* was calculated as $V_{z}=1/\left(n-3\right)$. Spearman correlations were analyzed as reported, consistent with evidence that pooling Pearson and Spearman correlations has minimal impact in this context [29].

We performed random-effects meta-analyses using robust variance estimation (RVE) to account for within-study dependence and to obtain cluster-robust standard errors without specifying an intraclass correlation structure [23,24,25,26]. Because the number of study clusters was small, we applied Tipton’s small-sample adjustment with Satterthwaite degrees of freedom for statistical inference [24]. Between-study heterogeneity was summarized using $\tau^{2}$ and $I^{2}$. We reported 95% confidence intervals (CIs) and 95% prediction intervals (PIs).

Prespecified subgroup analyses summarized pooled correlations by clinical scale (MTS vs. MAS), measurement position (STRETCH vs. REST), limb segment (upper vs. lower), and stroke chronicity (subacute vs. chronic). A restricted multivariable robust-variance meta-regression jointly evaluated scale, position, and output metric; limb segment and chronicity were explored in sensitivity analyses, consistent with a small-sample setting [23,24,25,26].

Small-study effects were examined using contour-enhanced funnel plots, a cluster-robust Egger test, and trim-and-fill. We also conducted influence diagnostics (including Baujat plots) and sensitivity analyses using alternative dependence assumptions (single-effect selection per study and within-study averaging with ρ=0.5 and ρ=0.8).

All analyses were performed in R (version 4.3.2) using robumeta, clubSandwich, and metafor packages [25,26,30]. Figures were generated using Python (version 3.14).

## 3. Results

### 3.1. Study Selection and Study Characteristics

**Figure 1:**
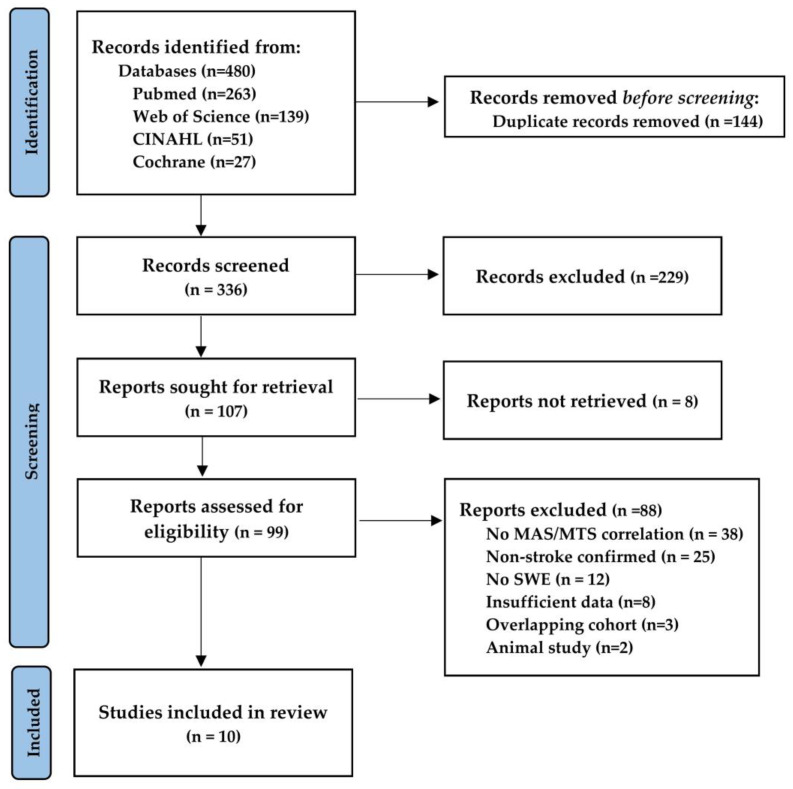
PRISMA 2020 flow diagram of the literature search and study selection process. Database searches (PubMed/MEDLINE, Web of Science Core Collection, CINAHL, and the Cochrane Library; inception to December 2025) identified 480 records; 144 duplicates were removed. After title/abstract screening of 336 records and full-text review of 99 articles, 10 studies were included in the quantitative synthesis (38 effect sizes). Reasons for full-text exclusion are shown in the diagram.

A total of 480 records were identified through database searching. After removal of duplicates, 336 unique titles and abstracts were screened, and 99 full-text articles were assessed for eligibility. Ten studies (N = 303) met the inclusion criteria and were included in the quantitative synthesis [31,32,33,34,35,36,37,38,39,40], contributing 38 correlation estimates across 10 study clusters (Figure 1).

During full-text review, an earlier publication from a cohort that was subsequently reported in expanded form was excluded to avoid double counting; the more comprehensive report was retained. Most included studies were published between 2017 and 2023 and were conducted in Asian countries. Upper-limb muscles—particularly the biceps brachii and brachialis—were most frequently examined, whereas lower-limb muscles (e.g., medial gastrocnemius) were less commonly studied. SWE outcomes were reported using either shear-wave velocity (m/s) or elastic modulus (kPa). Several studies contributed multiple correlations derived from different muscles, measurement positions, or clinical scales (Table 1).

### 3.2. Risk of Bias Assessment

According to the COSMIN methodology, overall construct-validity ratings were Adequate in 3 of 10 studies (30%), Doubtful in 4 of 10 studies (40%), and Inadequate in 3 of 10 studies (30%). Downgrading was most commonly driven by small sample sizes (*n* < 30 in 7 of 10 studies) and incomplete reporting of assessor blinding. Other COSMIN domains were generally rated as Adequate (Figure S1 and Table S2).

### 3.3. Overall Association Between SWE and Clinical Spasticity Measures

**Figure 2:**
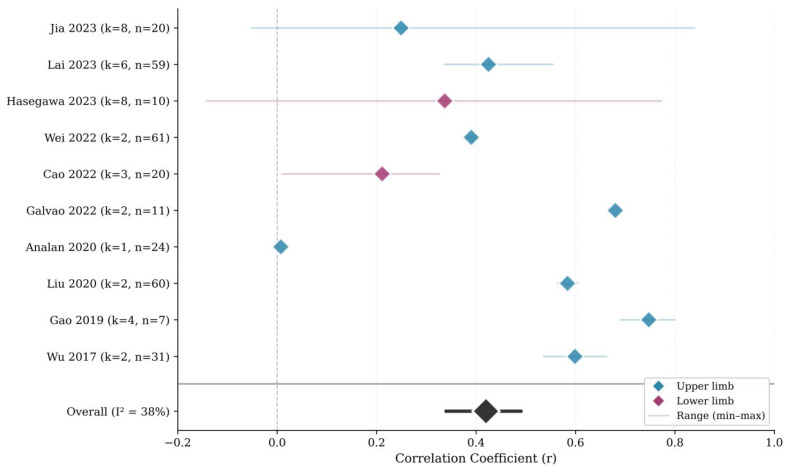
Study-level summary of the association between SWE and clinical spasticity ratings across the included studies [31,32,33,34,35,36,37,38,39,40]. For each study, the marker represents the mean correlation coefficient (*r*) aggregated across multiple dependent effect sizes, and the horizontal line indicates the within-study range (minimum to maximum *r*). The overall pooled estimate (*r* = 0.42, 95% CI 0.34–0.49; *I*^2^ = 38%) was obtained using robust variance estimation to account for within-study dependence. Studies are ordered chronologically. Blue markers denote upper-limb studies, purple markers denote lower-limb studies, and the black diamond represents the overall pooled estimate. *k* indicates the number of effect sizes and *n* the number of participants.

The pooled correlation between SWE-derived muscle stiffness and clinical spasticity ratings was *r* = 0.42 (95% CI, 0.34–0.49), with low-to-moderate heterogeneity (*I*^2^ = 38%). The 95% prediction interval ranged from 0.11 to 0.66, indicating that future studies conducted in comparable settings could plausibly observe correlations from small to large magnitudes (Figure 2). To ensure transparency and avoid selective reporting, the complete forest plot displaying all 38 individual effect sizes included in the robust variance meta-analysis is provided in Figure S2.

### 3.4. Subgroup Analyses

Subgroup analyses demonstrated higher pooled correlations when SWE was compared with the Modified Tardieu Scale (MTS) than with the Modified Ashworth Scale (MAS) (*r* = 0.49 vs. 0.39). Correlations were also higher when measurements were obtained in a stretched position (STRETCH) compared with a resting position (REST) (*r* = 0.49 vs. 0.38), with a trend toward significance in the between-group comparison (*p* ≈ 0.08).

By limb segment, correlations were higher for upper-limb muscles than for lower-limb muscles (*r* = 0.44 vs. 0.30). Pooled correlations were similar between subacute and chronic stroke populations (*r* = 0.41 vs. 0.46). These subgroup patterns were consistent with results from multivariable analyses (Table 2 and Figure S3).

### 3.5. Multivariable Meta-Regression

In the restricted multivariable meta-regression model including clinical scale, measurement position, and SWE output metric, the overall model reached statistical significance (QM = 8.4, *p* = 0.04). Given the limited number of independent study clusters (*m* = 10), these findings should be interpreted as exploratory.

Clinical scale was a significant moderator, with higher correlations observed for MTS compared with MAS (*β* = 0.168, *p* = 0.042). Measurement position showed a directionally consistent but non-significant effect favoring STRETCH over REST (*β* = 0.20, *p* = 0.10). The SWE output metric (kPa vs. m/s) was not associated with differences in correlation magnitude (*p* = 0.46).

Residual heterogeneity was low (*τ*^2^ = 0.015; *I*^2^ = 28%). Sensitivity models additionally including limb segment and stroke chronicity yielded similar results, with neither limb segment (*p* = 0.351) nor chronicity (*p* = 0.987) acting as significant moderators (Table S3).

### 3.6. Publication Bias and Robustness Analyses

**Figure 3:**
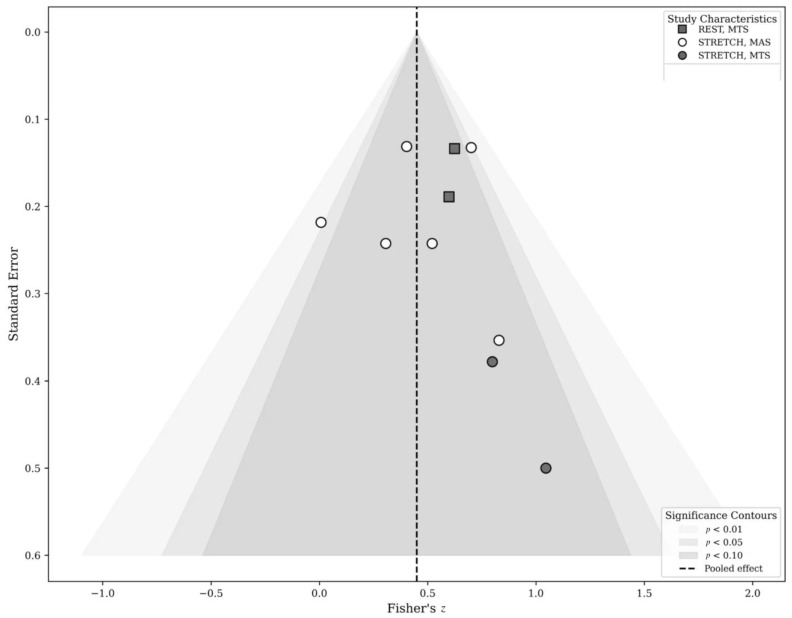
Funnel plot of the association SWE and clinical spasticity ratings. Each point represents an individual effect size expressed on Fisher’s *z* scale, plotted against its standard error. The vertical dashed line indicates the pooled effect estimate derived from the robust variance meta-analysis. Shaded contours denote regions of statistical significance (*p* < 0.10, *p* < 0.05, and *p* < 0.01). Visual inspection did not reveal marked funnel asymmetry, suggesting no strong evidence of small-study effects. Given the limited number of included studies, however, these findings should be interpreted cautiously. Symbol shapes indicate assessment condition (REST vs. STRETCH) and clinical scale (MAS vs. MTS).

Visual inspection of contour-enhanced funnel plots suggested no substantial asymmetry (Figure 3). Egger’s regression test was not statistically significant (*p* = 0.28), and trim-and-fill analysis did not impute any missing studies. Leave-one-out analyses yielded pooled correlations ranging from *r* = 0.40 to 0.45 (Table S4). Baujat diagnostics indicated low-to-moderate influence of individual studies (Figure S4).

Excluding studies with Inadequate COSMIN ratings resulted in a pooled correlation of *r* = 0.40 (95% CI, 0.31–0.49). Sensitivity analyses applying alternative dependency assumptions produced pooled estimates ranging from *r* = 0.40 to 0.48, with a maximum deviation of *Δr* = +0.06 under per-study selection of the strongest correlation (Table S5).

## 4. Discussion

### 4.1. Principal Findings

In this systematic review and meta-analysis of 10 studies, we found a moderate association between SWE-derived muscle stiffness and clinician-rated spasticity (*r* = 0.42), with low-to-moderate heterogeneity (*I*^2^ = 38%). This indicates that SWE captures a meaningful, but incomplete, proportion of the variance in clinical spasticity ratings [17,18,19,20,21,22]. Accordingly, SWE should be viewed as a quantitative adjunct, rather than a stand-alone substitute for established clinical grading systems [4,5,6].

Spasticity is defined as a velocity-dependent tonic stretch reflex resulting from supraspinal disinhibition and motor neuron hyperexcitability—a fundamentally neural phenomenon [3]. In contrast, SWE quantifies passive viscoelastic and structural tissue properties under static or quasi-static conditions. Because SWE does not directly assess reflex hyperexcitability, a complete correspondence with clinical scales that incorporate neural components is not expected. This physiological distinction helps explain the moderate magnitude of association observed in the present analysis.

Among the prespecified factors, clinical scale selection and measurement position were the most influential moderators of the SWE–spasticity association. In contrast, the SWE output metric, limb segment, and stroke chronicity did not significantly modify the observed correlations, although the available evidence—particularly for lower-limb muscles—was limited [12,13,14,15,16,31,32,33,34,35,36,37,38,39,40].

### 4.2. Influence of Clinical Scale (MTS vs. MAS)

Correlations were higher when SWE was compared with the MTS than with the MAS, and this difference became statistically significant after adjustment for measurement position and output metric. This finding is clinically plausible. The MAS provides a single ordinal score that combines neural and passive components without velocity standardization and demonstrates only moderate inter-rater reliability [4,5]. In contrast, the MTS applies a standardized fast stretch and separately records the dynamic catch (R1) and the passive range (R2), offering a clearer separation of reflex-mediated and passive components [4,6].

Because SWE is typically acquired under relaxed conditions and primarily reflects passive mechanical stiffness, it aligns more closely with the passive elements captured by the MTS. Although fewer studies reported MTS-based correlations, the statistical strength of the scale effect in the multivariable model supports prioritizing the MTS over the MAS when validating quantitative stiffness measures. Nevertheless, this finding should be confirmed in adequately powered head-to-head studies collecting both scales under identical SWE protocols.

### 4.3. Influence of Measurement Position (STRETCH vs. REST)

Measurement position showed a consistent pattern, with higher correlations observed when SWE was performed in stretched rather than resting positions. Although this effect did not reach statistical significance, the direction and magnitude were consistent across analyses.

This pattern is biomechanically plausible. Measurements obtained at rest may be influenced by tissue slack and low passive tension, reducing sensitivity to stiffness differences, whereas stretched positions increase passive tension and improve discrimination along the muscle length–tension relationship [12,13,14,15,16]. From a practical standpoint, these findings support standardizing stretched positions to improve protocol consistency and alignment with clinical assessment, rather than assuming that any single position will universally maximize correlations.

### 4.4. Output Metric, Limb Segment, and Chronicity

The choice of SWE output metric did not meaningfully influence observed associations, which is expected given the close mathematical relationship between shear-wave velocity and elastic modulus [7,8,9,10,11]. Reporting shear-wave speed (m/s) as the primary metric may therefore improve comparability across studies, with elastic modulus reported secondarily when appropriate.

Limb segment and stroke chronicity were not significant moderators. However, the evidence base for lower-limb muscles was limited, and correlations for the lower limb were numerically smaller. This may reflect a mismatch between typical SWE acquisition conditions (supine, quasi-static) and the functional relevance of lower-limb spasticity during gait and weight-bearing activities [41,42]. In addition, anatomical and architectural differences may influence SWE performance in the lower extremity. Lower-limb muscles such as the gastrocnemius are typically deeper and exhibit greater pennation angles compared with the more superficial upper-limb muscles predominantly studied [14,15,16]. Greater depth may reduce signal quality, potentially affecting measurement reliability [8,11]. Moreover, because SWE-derived modulus assumes uniform tissue behavior [7,8], highly pennate muscle architecture may introduce directional variability in stiffness estimates [9,14]. These structural characteristics may partly explain the comparatively lower correlations observed in lower-limb analyses.

Similarly, the absence of a chronicity effect may reflect the presence of passive tissue stiffening across recovery stages [3], but longitudinal data are needed to clarify time-dependent patterns.

### 4.5. Relation to Previous Literature

Previous reviews across mixed neurological populations have reported wide variability in SWE–spasticity associations [18,19,20,21,22]. Such variability is expected given differences in diagnoses, target muscles, and acquisition protocols. By restricting the analysis to adults with stroke, the present study demonstrates a more consistent association with lower heterogeneity, supporting the value of diagnostic homogeneity for quantitative synthesis.

This study also extends prior work by addressing within-study statistical dependence using RVE and by formally evaluating protocol-related factors that are directly relevant to clinical practice [23,24,25,26]. Rather than treating protocol variation as unexplained noise, our findings highlight specific choices—particularly scale selection and measurement position—that can meaningfully influence the magnitude of SWE–spasticity associations.

### 4.6. Clinical Implications

These findings support the use of SWE as a complementary tool in post-stroke spasticity assessment:

- Clinical scale: When feasible, pairing SWE with the MTS may improve interpretability, while the MAS may be retained for continuity [4,5,6].
- Measurement position: Standardizing stretched positions and clearly documenting joint angles can improve reproducibility [12,13,14,15,16].
- Output metric: Reporting shear-wave speed (m/s) as the primary outcome may facilitate comparison across studies [7,8,9,10,11].
- Limb segment: Current evidence is strongest for upper-limb assessment; lower-limb applications remain important but less well studied [31,33,35,38,39,40,41,42].
- Chronicity: SWE appears informative in both subacute and chronic stages and may be considered when clinically indicated [3].

Importantly, SWE values should not be interpreted using fixed cut-offs corresponding to clinical grades. Instead, SWE is best suited for tracking within-patient change over time alongside standardized clinical assessment [6].

### 4.7. Limitations

Several limitations warrant consideration. The overall evidence base was modest, limiting statistical precision for subgroup and moderator analyses, particularly for lower-limb muscles [16,32,35]. Although robust variance estimation with small-sample correction was applied, the limited number of independent study clusters (m = 10) constrains the stability of multivariable meta-regression findings; these results should therefore be interpreted as exploratory and hypothesis-generating rather than definitive.

A conceptual distinction also exists between what SWE measures and the clinical definition of spasticity. SWE does not directly assess reflex hyperexcitability—the hallmark neural feature of spasticity [3,43]—but instead quantifies its mechanical consequences, specifically increased passive muscle stiffness. This distinction is particularly relevant when interpreting MTS findings, as R1 reflects the velocity-dependent neural “catch”, whereas R2 represents passive end-range resistance associated with structural tissue properties [4]. In the present review, correlations were pooled across R1, R2, and derived indices (e.g., R2−R1) because most studies did not report these components separately. Given that SWE assesses tissue stiffness while the muscle is held at rest or moved slowly, it would be expected to align more closely with R2 than with R1. However, only one included study (Hasegawa et al., 2023 [35]) reported R1- and R2-specific correlations independently, precluding formal component-specific meta-analysis. Future studies should report these components separately to clarify the relative neural and structural contributors to SWE-derived stiffness.

Measurement reproducibility also warrants consideration. SWE values are sensitive to patient posture, muscle activation state, and operator technique, yet these acquisition parameters were incompletely described in several reports [8,9,10,11,18,19,20,21,22]. Such variability may limit comparability across studies and generalizability across clinical settings. Furthermore, lower-limb muscles are typically deeper and more pennate than upper-extremity muscles. Because SWE-derived elastic modulus assumes isotropic tissue behavior [7,8], architectural complexity may introduce systematic estimation error and may partly explain the lower correlations observed in lower-limb investigations.

Finally, most included studies were cross-sectional in design, precluding causal or prognostic inference [43,44]. Although no strong evidence of publication bias was detected, the limited number of studies and their concentration within a small number of geographic regions warrant cautious interpretation when generalizing these findings to broader healthcare systems [45].

### 4.8. Future Directions

Future research should prioritize protocol standardization, adequately powered head-to-head comparisons of clinical scales, and expansion to lower-limb and functionally relevant assessment conditions [12,13,14,15,16,41,42]. Longitudinal studies are needed to evaluate responsiveness over time, and integration with instrumented biomechanical measures may help clarify how passive stiffness contributes to clinically observed resistance [6,43,44]. Establishing clinically meaningful change thresholds will be essential to support routine clinical adoption [6,18,19,20,21,22].

## 5. Conclusions

Current evidence indicates that SWE reflects the mechanical consequences of post-stroke spasticity and shows a moderate association with clinician-rated spasticity scales. These findings suggest that SWE and clinical scales assess related but not identical constructs. Accordingly, SWE should be regarded as a quantitative adjunct reflecting passive muscle stiffness rather than a direct measure of neural spasticity. Stronger associations were observed when SWE was paired with the MTS and when measurements were obtained under standardized stretched conditions. Evidence is more consistent for upper-limb muscles, while lower-limb applications remain limited and require further validation. Future studies should prioritize adequately powered lower-limb investigations and focus on defining clinically interpretable thresholds and minimally clinically important difference values to facilitate routine clinical use.

## Figures and Tables

**Table 1:** Characteristics of studies included in the meta-analysis.

Author	Year	Country	Study Design	N	Mean Age	Chronicity	Muscle(s) ^a^	Position ^b^	Scale ^c^	Metric	Effect Sizes (k) ^d^
Analan et al.	2020	Turkey	Cross-sectional	24	57.5	Chronic	BB	STRETCH	MAS	m/s	1
Cao et al.	2022	China	Cross-sectional	20	52.3	Subacute	MG	Both	MAS	kPa	3
Galvao et al.	2022	Brazil	Cross-sectional	11	55.6	Chronic	BB, BR	STRETCH	MAS	kPa	2
Gao et al.	2019	USA	Single arm pre-post (baseline only)	7	58.0	Chronic	BB	Both	MAS; MTS	m/s	4
Hasegawa et al.	2023	Japan	Single arm pre-post (baseline only)	10	62.7	Chronic	MG	Both	MAS; MTS	m/s	8
Jia et al.	2023	China	Cross-sectional	20	53.9	Subacute	AD, LD, PM, TM	Both	MAS	kPa	8
Lai et al.	2023	Taiwan	Cross-sectional	59	55.7	Subacute	FCR, FCU, FDS	REST	MAS; MTS	m/s	6
Liu et al.	2020	China	Single arm pre-post (baseline only)	60	66.0	Subacute	BB	STRETCH	MAS	m/s kPa	2
Wei et al.	2022	China	Cross-sectional	61	63.5	Subacute	BB	STRETCH	MAS	m/s	2
Wu et al.	2017	Taiwan	Cross-sectional	31	60.3	Subacute	BB	REST	MAS; MTS	m/s	2

Abbreviations: AD = anterior deltoid; BB = biceps brachii; BR = brachioradialis; FCU = flexor carpi ulnaris; FCR = flexor carpi radialis; FDS = flexor digitorum superficialis; LD = latissimus dorsi; MAS = Modified Ashworth Scale; MG = medial gastrocnemius; MTS = Modified Tardieu Scale; PM = pectoralis major; TM = teres major; m/s = shear-wave speed; kPa = Young’s modulus. ^a^ Multiple muscles assessed within the same participant cohort contributed separate effect sizes to the meta-analysis. ^b^ “Both” indicates that correlations were reported for both REST and STRETCH measurement conditions. ^c^ For MTS, studies reported correlations using one or more components (R1, R2, R2–R1) or angle-based indices. ^d^ Effect sizes were analyzed on Fisher’s *z* scale and back-transformed to Pearson’s *r* for reporting.

**Table 2:** Pooled correlations between SWE-derived muscle stiffness and clinical spasticity ratings.

Subgroup	m	k	*r*	95% CI	95% PI	*I* ^2^	Q Between	*p*-Value
Scale								
MAS	10	26	0.39	[0.28, 0.49]	[−0.01, 0.68]	51%	2.27	0.13
MTS	4	12	0.49	[0.38, 0.59]	[0.38, 0.59]	0%		
Measurement Position								
REST	6	20	0.38	[0.28, 0.47]	[0.18, 0.54]	19%	2.99	0.08
STRETCH	8	18	0.49	[0.35, 0.61]	[0.06, 0.76]	49%		
Metric								
m/s	7	24	0.43	[0.32, 0.53]	[0.03, 0.72]	45%	0.90	0.34
kPa	4	14	0.40	[0.25, 0.53]	[0.18, 0.58]	16%		
Limb								
Upper	8	27	0.45	[0.35, 0.53]	[0.12, 0.69]	43%	2.70	0.10
Lower	2	11	0.30	[0.09, 0.48]	[0.09, 0.48]	0%		
Chronicity								
Subacute	6	23	0.41	[0.31, 0.50]	[0.08, 0.67]	48%	0.01	0.92
Chronic	4	15	0.46	[0.28, 0.61]	[0.14, 0.69]	11%		
Overall	10	38	0.42	[0.34, 0.49]	[0.11, 0.66]	38%		

Abbreviations: CI, confidence interval; PI, prediction interval; SWE, shear-wave elastography. Notes: Random-effects models were estimated using RVE with Tipton’s small-sample correction to account for dependent effect sizes within studies. Between-group *p* values were obtained from RVE-based single-moderator tests with cluster-RVE. Effect sizes were analyzed on Fisher’s *z* scale and back-transformed to Pearson’s *r* for presentation. The 95% PI represents the expected range of the true correlation in a comparable future study. *m* denotes the number of contributing studies and *k* the number of effect sizes; *m* does not sum across subgroups because individual studies may contribute effect sizes to multiple strata.

## Data Availability

The data analyzed in this study were extracted from previously published articles. The extracted datasets and the analytic code used to support the findings of this study are available from the corresponding author upon reasonable request.

## Supplementary Materials

The following supporting information can be downloaded at: https://www.mdpi.com/article/10.3390/jcm15052063/s1. Table S1: Database-specific search strategies; Table S2: COSMIN ratings for construct validity (hypotheses testing domain); Table S3: Multivariable meta-regression using robust variance estimation; Table S4: Leave-one-study-out sensitivity analysis of the pooled correlation; Table S5: Sensitivity analyses for pooled correlation estimates; Table S6: PRISMA 2020 Checklist. Figure S1: Risk of bias assessment of included studies using the COSMIN methodology; Figure S2: Comprehensive forest plot of individual correlations between SWE and clinical spasticity ratings; Figure S3: Subgroup analyses of the association between SWE and clinical spasticity ratings; Figure S4: Baujat plot assessing study influence and contribution to heterogeneity.

## Abbreviations

The following abbreviations are used in this manuscript:

SWE	Shear-wave elastography
MAS	Modified Ashworth Scale
MTS	Modified Tardieu Scale
RVE	Robust variance estimation
PRISMA	Preferred Reporting Items for Systematic Reviews and Meta-Analyses
COSMIN	Consensus-based Standards for the selection of health Measurement Instruments
CI	Confidence interval
PI	Prediction interval
