# Supplementary material for: Clinical Validity of Shear Wave Elastography for Post-Stroke Spasticity: A Systematic Review and Meta-Analysis
Source: J Clin Med. 2026 Mar 9;15(5):2063. doi: 10.3390/jcm15052063 (PMC12985620; doi:10.3390/jcm15052063)
Supplement: Supplementary file 1 [file jcm-15-02063-s001.zip › jcm-4160251-supplementary/jcm-4160251-supplementary.pdf]

## Supplementary Material

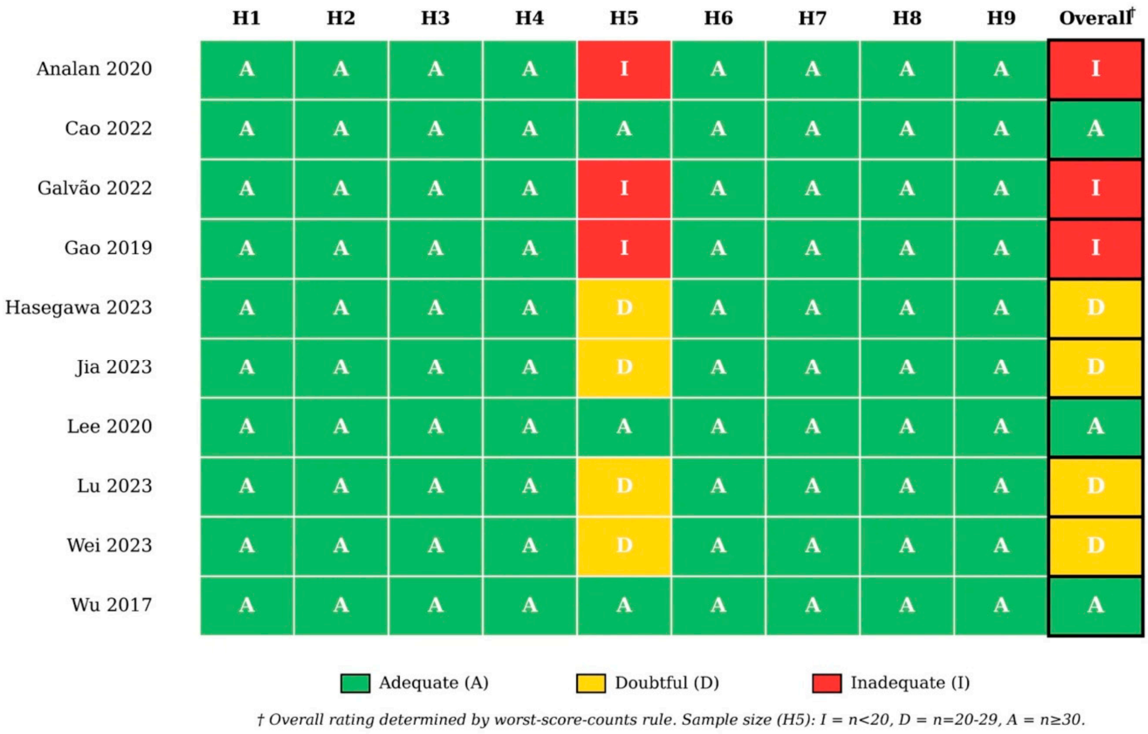


**Figure S1. Risk of bias assessment of included studies using the COSMIN methodology.** Overall construct validity was evaluated according to the COSMIN (Consensus-based Standards for the selection of health Measurement Instruments) framework. Each study was rated across predefined construct-validity domains and classified as Adequate, Doubtful, or Inadequate based on the worst-score-counts principle. Downgrading was most commonly driven by small sample sizes and incomplete reporting of assessor blinding, whereas other domains were generally rated as Adequate. Detailed item-level ratings are provided in Supplementary Table S2.

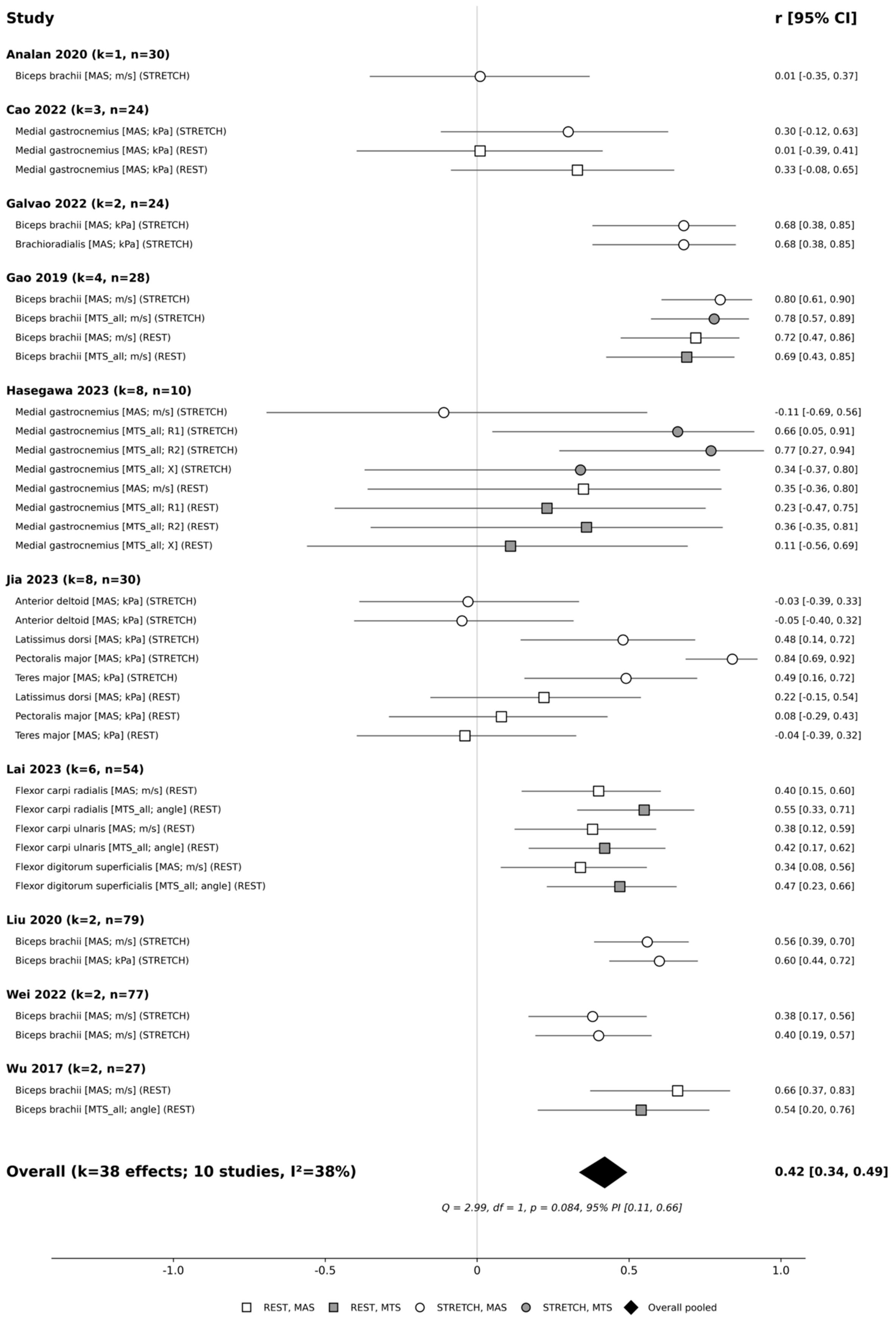


**Figure S2. Comprehensive forest plot of individual correlations between SWE and clinical spasticity ratings.** This forest plot displays all 38 individual correlation coefficients extracted from the 10 included studies. Each marker represents a single effect size derived from a specific muscle, measurement position (STRETCH or REST), clinical scale (MAS or MTS), and output metric (m/s or kPa), with horizontal lines indicating the corresponding 95% confidence intervals. Studies contributing multiple effect sizes are grouped together, reflecting within-study dependence. The overall pooled estimate (diamond) was calculated using random-effects meta-analysis with RVE to account for dependent effect sizes. Square markers denote REST measurements, and circular markers denote STRETCH measurements; open symbols indicate MAS and filled symbols indicate MTS. The vertical line at $r = 0$ indicates no association.

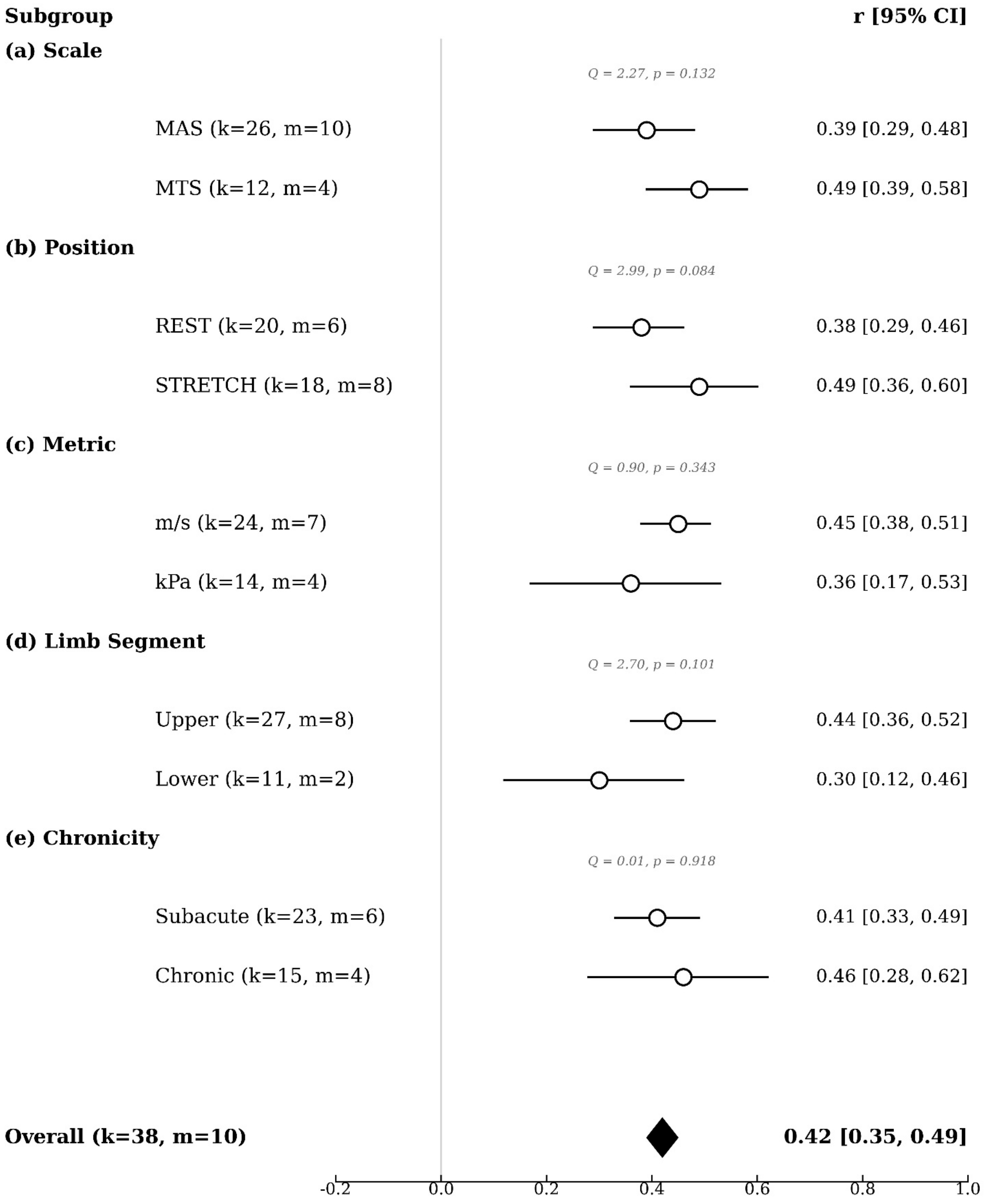


**Figure S3. Subgroup analyses of the association between SWE and clinical spasticity ratings.** Forest plots showing pooled correlation coefficients (*r*) and 95% confidence intervals for prespecified subgroups according to (a) clinical scale (MAS vs. MTS), (b) measurement position (REST vs. STRETCH), (c) output metric (m/s vs. kPa), (d) limb segment (upper vs. lower), and (e) chronicity (subacute vs. chronic). Subgroup estimates were obtained using random-effects models with RVE to account for dependent effect sizes within studies. Between-subgroup differences were evaluated using RVE-based *Q* statistics, with corresponding *p* values shown for each moderator. The overall pooled estimate across all subgroups is displayed at the bottom. *k* denotes the number of effect sizes and *m* the number of contributing studies.

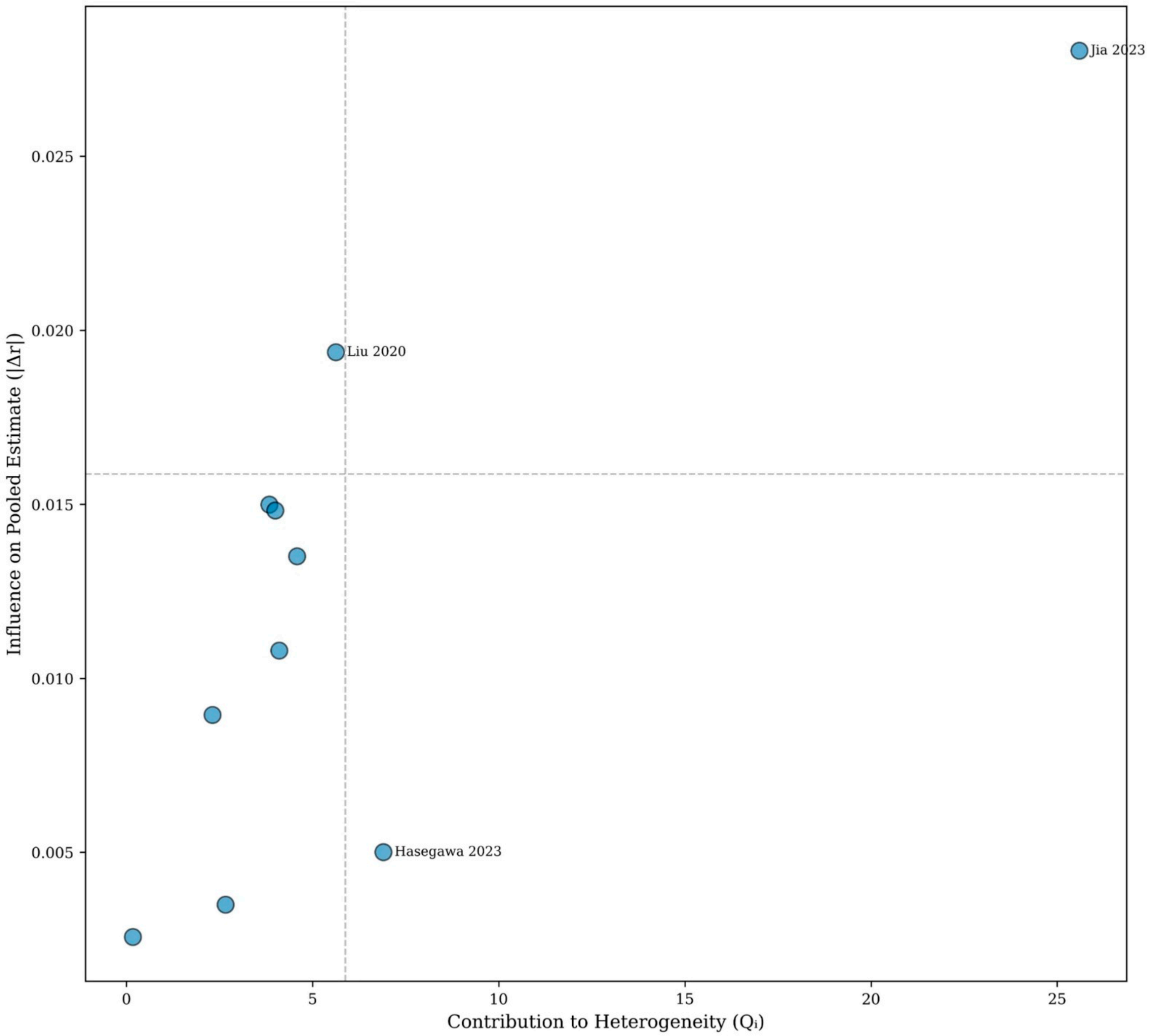


**Figure S4. Baujat plot assessing study influence and contribution to heterogeneity.** The Baujat plot displays each study's contribution to overall heterogeneity (Q statistic, x-axis) against its influence on the pooled effect estimate ($\Delta\tau^2$, y-axis). Studies located toward the upper-right quadrant contribute more strongly to both heterogeneity and influence on the pooled correlation. No single study exerted disproportionate influence on the overall estimate, and the primary findings remained robust across sensitivity analyses.

**Table S1. Database-specific search strategies**

| Database | Search Strategy |
|---|---|
| **Pubmed** | #1 SWE/Elastography Terms<br><br>"Elasticity Imaging Techniques"[Mesh] OR ((elastograph*[tiab] OR "shear wave"[tiab] OR shear-wave[tiab] OR SWE[tiab] OR "acoustic radiation force impulse"[tiab] OR ARFI[tiab] OR "supersonic shear imaging"[tiab] OR SSI[tiab] OR "point shear wave"[tiab] OR pSWE[tiab] OR "shear modulus"[tiab] OR stiffness[tiab] OR "Young's modulus"[tiab]) AND (ultrasound[tiab] OR ultrasonograph*[tiab] OR sonograph*[tiab] OR "Ultrasonography"[Mesh]))<br><br>#2 Spasticity/Hypertonia Terms<br><br>"Muscle Hypertonia"[Mesh] OR spastic*[tiab] OR hypertonia[tiab] OR "muscle tone"[tiab] OR "muscle stiffness"[tiab] OR "upper motor neuron"[tiab]<br><br>#3 Clinical Scales and Validity Terms<br><br>"Ashworth Scale"[Mesh] OR "modified ashworth"[tiab] OR "ashworth scale"[tiab] OR MAS[tiab] OR tardieu[tiab] OR "modified tardieu"[tiab] OR MTS[tiab] OR correlat*[tiab] OR validity[tiab] OR "construct validity"[tiab] OR convergent[tiab] OR concurrent[tiab] OR criterion[tiab] OR agreement[tiab] OR spearman[tiab] OR pearson[tiab]<br><br>#4 Exclusions<br><br>NOT ("magnetic resonance"[tiab] OR MRE[tiab])<br><br>FINAL: #1 AND #2 AND #3 AND #4 |
| **Cochrane Library** | #1 SWE/Elastography Terms<br><br>[mh "Elasticity Imaging Techniques"] OR ((elastograph* OR "shear wave" OR SWE OR ARFI OR "supersonic shear" OR "point shear" OR "shear modulus" OR stiffness) NEAR/3 (ultrasound OR ultrasonograph* OR sonograph*)):ti,ab,kw<br><br>#2 Spasticity/Hypertonia Terms<br><br>[mh "Muscle Hypertonia"] OR (spastic* OR hypertonia OR "muscle tone" OR "muscle stiffness" OR "upper motor neuron"):ti,ab,kw<br><br>#3 Clinical Scales and Validity Terms<br><br>[mh "Ashworth Scale"] OR ("modified ashworth" OR "ashworth scale" OR MAS OR tardieu OR "modified tardieu" OR MTS):ti,ab,kw OR (correlat* OR validity OR "construct validity" OR convergent OR concurrent OR criterion OR agreement OR spearman OR pearson):ti,ab,kw<br><br>#4 Exclusions<br><br>NOT (("magnetic resonance" NEAR/1 elastograph*):ti,ab,kw)<br><br>FINAL: #1 AND #2 AND #3 AND #4 |

| | |
|---|---|
| **CINAHL (EBSCOhost)** | #1 SWE/Elastography Terms<br><br>(MH "Elasticity Imaging Techniques+") OR ((elastograph* OR "shear wave" OR SWE OR ARFI OR "supersonic shear" OR "point shear" OR "shear modulus" OR stiffness) N3 (ultrasound OR ultrasonograph* OR sonograph*))<br><br>#2 Spasticity/Hypertonia Terms<br><br>(MH "Muscle Hypertonia+") OR spastic* OR hypertonia OR "muscle tone" OR "muscle stiffness" OR "upper motor neuron"<br><br>#3 Clinical Scales and Validity Terms (MH "Ashworth Scale") OR ("modified ashworth" OR "ashworth scale" OR MAS OR tardieu OR "modified tardieu" OR MTS) OR (correlat* OR validity OR "construct validity" OR convergent OR concurrent OR criterion OR agreement OR spearman OR pearson)<br><br>#4 Exclusions<br><br>NOT ("magnetic resonance" N1 elastograph*)<br><br>FINAL: #1 AND #2 AND #3 AND #4 |
| **Web of Science (Core Collection)** | #1 SWE/Elastography Terms<br><br>((ultrasound OR ultrasonograph* OR sonograph*) NEAR/3 (elastograph* OR "shear wave" OR SWE OR ARFI OR "acoustic radiation force impulse" OR "supersonic shear" OR SSI OR "point shear" OR pSWE OR "shear modulus" OR stiffness OR "Young's modulus"))<br><br>#2 Spasticity/Hypertonia Terms<br><br>(spastic* OR hypertonia OR "muscle tone" OR "muscle stiffness" OR "upper motor neuron")<br><br>#3 Clinical Scales and Validity Terms<br><br>("modified ashworth" OR "ashworth scale" OR MAS OR tardieu OR "modified tardieu" OR MTS OR correlat* OR validity OR "construct validity" OR convergent OR concurrent OR criterion OR agreement OR spearman OR pearson)<br><br>#4 Exclusions<br><br>NOT (("magnetic resonance" NEAR/1 elastograph*) OR MRE OR liver OR hepatic OR thyroid OR breast OR prostate OR pancreas OR kidney OR renal)<br><br>FINAL: #1 AND #2 AND #3 AND #4 |

**Abbreviations:** ARFI, acoustic radiation force impulse; MAS, Modified Ashworth Scale; Mesh/MH, Medical Subject Headings; MRE, magnetic resonance elastography; MTS, Modified Tardieu Scale; pSWE, point shear-wave elastography; SSI, supersonic shear imaging; SWE, shear-wave elastography; tiab, title/abstract; kw, keyword. / **Notes:** Database-specific search strategies were developed and adapted for each platform to identify studies evaluating the association between SWE–derived muscle stiffness and clinical spasticity measures after stroke. Controlled vocabulary (e.g., MeSH or CINAHL Headings) and free-text terms were combined using Boolean operators. Proximity operators (e.g., NEAR/n, Nn) were applied where supported. No language or publication date restrictions were applied at the search stage. Searches were conducted from database inception to December 2025. The complete search strategy for each database is reported to ensure transparency and reproducibility.

**Table S2. COSMIN ratings for construct validity (hypotheses testing domain)**

| Study | H1 | H2 | H3 | H4 | H5 | H6 | H7 | H8 | H9 | Overall Rating |
|---|---|---|---|---|---|---|---|---|---|---|
| *Analan et al.*, 2020 | A | A | A | A | I | A | A | A | A | Inadequate |
| *Cao et al.*, 2022 | A | A | A | A | A | A | A | A | A | Adequate |
| *Galvao et al.*, 2022 | A | A | A | A | I | A | A | A | A | Inadequate |
| *Gao et al.*, 2019 | A | A | A | A | I | A | A | A | A | Inadequate |
| *Hasegawa et al.*, 2023 | A | A | A | A | D | A | A | A | A | Doubtful |
| *Jia et al.*, 2023 | A | A | A | A | D | A | A | A | A | Doubtful |
| *Lai et al.*, 2023 | A | A | A | A | A | A | A | A | A | Adequate |
| *Liu et al.*, 2020 | A | A | A | A | D | A | A | A | A | Doubtful |
| *Wei et al.*, 2022 | A | A | A | A | D | A | A | A | A | Doubtful |
| *Wu et al.*, 2017 | A | A | A | A | A | A | A | A | A | Adequate |

**Abbreviations:** COSMIN, Consensus-based Standards for the selection of health Measurement Instruments; SWE, shear-wave elastography; V, Very good; A, Adequate; D, Doubtful; I, Inadequate.

**Notes:** Construct validity was assessed using the COSMIN methodology for hypotheses testing. Each study was evaluated across nine predefined items (H1–H9) using a four-point rating scale (Very good, Adequate, Doubtful, or Inadequate). Overall study quality was determined according to the worst-score-counts principle, whereby the lowest rating across items H1–H9 defines the overall rating.

Item definitions were as follows:
H1, formulation of a priori hypotheses;
H2, adequacy of the comparator instrument;
H3, appropriateness of study design;
H4, adequacy of statistical methods;
H5, sample size;
H6, handling of missing data;
H7, absence of other important methodological flaws;
H8, completeness of results reporting;
H9, assessor blinding.

The following operational decisions were prespecified in the review protocol:
(1) Sample size (H5) was rated Adequate for ≥30 participants, Doubtful for 20–29 participants, and Inadequate for <20 participants.
(2) Assessor blinding (H9) was rated Adequate when blinding was not reported, given the objective, device-generated nature of SWE measurements, whereas explicitly unblinded assessments were rated Doubtful.

**Table S3. Multivariable meta-regression using robust variance estimation**

**Main Analysis (Scale, Position, Metric)**

| Moderator | β | SE | t | *Df* | p |
|---|---|---|---|---|---|
| Intercept (MAS, REST, m/s) | 0.345 | 0.084 | 4.11 | 6 | 0.006 |
| Scale (MTS vs MAS) | 0.168 | 0.065 | 2.57 | 6 | 0.042* |
| Position (STRETCH vs REST) | 0.203 | 0.106 | 1.93 | 6 | 0.102 |
| Metric (kPa vs m/s) | -0.077 | 0.099 | -0.78 | 6 | 0.464 |

**Sensitivity Analysis (Main + Limb, Chronicity)**

| Moderator | β | SE | t | *df* | p |
|---|---|---|---|---|---|
| Intercept (MAS, REST, m/s, Lower, Subacute) | 0.163 | 0.171 | 0.95 | 4 | 0.397 |
| Scale (MTS vs MAS) | 0.203 | 0.115 | 1.76 | 4 | 0.154 |
| Position (STRETCH vs REST) | 0.192 | 0.166 | 1.16 | 4 | 0.312 |
| Metric (kPa vs m/s) | -0.023 | 0.166 | -0.14 | 4 | 0.898 |
| Limb (Upper vs Lower) | 0.190 | 0.180 | 1.05 | 4 | 0.351 |
| Chronicity (Chronic vs Subacute) | -0.005 | 0.304 | -0.02 | 4 | 0.987 |

**Abbreviations:**: β, regression coefficient (difference in Fisher's *z*-transformed correlation relative to the reference category); SE, standard error; df, Satterthwaite degrees of freedom; MAS, Modified Ashworth Scale; MTS, Modified Tardieu Scale; SWE, shear-wave elastography.

**Notes:** Multivariable random-effects meta-regression models were estimated using RVE to account for dependent effect sizes within studies. Fisher's *z*-transformed correlation coefficients were used as the outcome and back-transformed to Pearson's *r* for presentation elsewhere. Small-sample inference was performed using Tipton's correction with Satterthwaite-adjusted degrees of freedom. In the main analysis, the reference category for the intercept was MAS, REST position, and m/s output metric. In the sensitivity analysis, the reference category was MAS, REST position, m/s metric, lower limb, and subacute stroke stage. Regression coefficients (β) represent the expected difference in Fisher's *z*-transformed correlation associated with each moderator relative to its

reference category. Given the limited number of study clusters, limb segment and chronicity were evaluated in sensitivity analyses rather than in the primary model. Statistical significance was assessed at a two-sided $\alpha$ level of 0.05; an asterisk (*) denotes $p < 0.05$.

**Table S4. Leave-one-study-out sensitivity analysis of the pooled correlation**

| Study Excluded | Effect Sizes (k) Remaining | r | 95% CI | Δr |
|---|---|---|---|---|
| *Analan et al.*, 2020 | 37 | 0.43 | [0.36, 0.50] | +0.01 |
| *Cao et al.*, 2022 | 35 | 0.43 | [0.35, 0.50] | +0.01 |
| *Galvao et al.*, 2022 | 36 | 0.41 | [0.33, 0.48] | −0.01 |
| *Gao et al.*, 2019 | 34 | 0.40 | [0.33, 0.47] | -0.02 |
| *Hasegawa et al.*, 2023 | 30 | 0.45 | [0.37, 0.52] | +0.03 |
| *Jia et al.*, 2023 | 30 | 0.45 | [0.38, 0.51] | +0.03 |
| *Lai et al.*, 2023 | 32 | 0.42 | [0.34, 0.50] | 0.00 |
| *Liu et al.*, 2020 | 36 | 0.40 | [0.33, 0.47] | −0.02 |
| *Wei et al.*, 2022 | 36 | 0.43 | [0.35, 0.50] | +0.01 |
| *Wu et al.*, 2017 | 36 | 0.40 | [0.33, 0.47] | −0.02 |

**Abbreviations:** CI, confidence interval; k, number of effect sizes; m, number of study clusters; RVE, robust variance estimation.

**Notes:** Leave-one-study-out analyses were conducted by sequentially excluding each study cluster and refitting random-effects models using RVE with Satterthwaite degrees of freedom. Δr denotes the change in the pooled correlation relative to the main analysis (r = 0.42, 95% CI 0.35–0.49; k = 38; m = 10). Exclusion of any single study did not shift the pooled estimate outside the 95% confidence interval of the main analysis.

**Table S5. Sensitivity analyses for pooled correlation estimates**

| Analysis | K | m | r [95% CI] | Δr |
|---|---|---|---|---|
| **Main Analysis (RVE)** | 38 | 10 | 0.42 [0.34, 0.49] | — |
| **Alternative Dependency Handling** | | | | |
| Rule-based selection (highest r per study) | 10 | 10 | 0.48 [0.34, 0.59] | +0.06 |
| Averaging (ϱ=0.5) | 10 | 10 | 0.44 [0.28, 0.57] | +0.02 |
| Averaging (ϱ=0.8) | 10 | 10 | 0.44 [0.28, 0.57] | +0.02 |
| **Quality-Based Exclusion** | | | | |
| Excluding Inadequate studies | 32 | 7 | 0.40 [0.31, 0.49] | −0.02 |
| **Leave-One-Out (range)** | - | 10 | 0.40–0.45 | ±0.03 |
| **Influence Diagnostics** | | | | |
| Max influence (Gao 2019) | | | | +0.03 |
| **Publication Bias** | | | | |
| Trim-and-fill (0 studies imputed) | 38 | 10 | 0.42 [0.35, 0.49] | 0.00 |

**Abbreviations:** CI, confidence interval; k, number of effect sizes; m, number of study clusters; RVE, robust variance estimation; ϱ, assumed within-study correlation

**Notes:** Δr indicates the change in the pooled correlation coefficient relative to the main analysis (r = 0.42 [95% CI, 0.34–0.49]). All meta-analytic models were estimated on Fisher's z scale and back-transformed to Pearson's r for presentation. Robust variance estimation (RVE) with Tipton's small-sample correction was used to account for within-study dependence. For averaging approaches, ϱ denotes the assumed within-study correlation. The trim-and-fill procedure did not impute any missing studies.
